# Activated phosphoinositide 3-kinase δ syndrome associated with nephromegaly, growth hormone deficiency, bronchiectasis: a case report

**DOI:** 10.1186/s13223-022-00655-5

**Published:** 2022-02-21

**Authors:** Megan Craig, Bob Geng, Kristen Wigby, Susan A. Phillips, Christine Bakhoum, John Naheedy, Mateja Cernelc-Kohan

**Affiliations:** 1 University of California San Diego, 9500 Gilman Drive, La Jolla, CA 92093 USA; 2 Rady Children’s Hospital, 3020 Children’s Way, #5124, San Diego, CA 92123 USA; 3 Rady Children’s Institute for Genomic Medicine, 7920 Frost Street, San Diego, CA 92123 USA; 4 Section of Pediatric Nephrology, Department of Pediatrics, Yale University, 333 Cedar Street, New Haven, CT 06520 USA

**Keywords:** Primary immunodeficiency, Bronchiectasis, Asthma, Pituitary abnormality, Growth hormone deficiency, Nephromegaly

## Abstract

**Background:**

Activated phosphoinositide 3-kinase (PI3K) δ syndrome (APDS) is a rare form of primary immunodeficiency with 243 known cases reported in the literature. Known findings associated with the condition include recurrent sinusitis and bronchitis, bronchiectasis, immune cytopenias, mild developmental delay, splenomegaly, and lymphadenopathy. We report the case of a child with APDS accompanied by unique clinical features: nephromegaly and growth hormone deficiency with associated pituitary anatomic abnormality.

**Case presentation:**

The patient is a nine-year-old boy with a heterozygous de novo variant in *phosphatidylinositol-4,5-bisphosphate 3-kinase catalytic subunit δ* (p.E1021K), previously reported in association with APDS. Our patient, who had no family history of immunodeficiency, exhibits classic findings of this syndrome but also has unique features that extend the phenotypic spectrum of this disorder. At 5 years of age, the patient showed marked growth deceleration and was demonstrated to have growth hormone (GH) deficiency with associated pituitary anatomic abnormality. He started GH therapy with an excellent response. He additionally has bilateral nephromegaly of unclear etiology, microscopic hematuria and proteinuria, asthma, and has developed left hip pain with arthrocentesis consistent with oligoarticular juvenile idiopathic arthritis. At age nine, the patient was referred to genetics and whole exome sequencing revealed APDS. Though there was initial concern that GH may increase risk for malignancy as GH signals through the PI3K pathway, he was allowed to continue treatment as the PI3K pathway was considered constitutively active at baseline.

**Conclusions:**

Our patient’s unique presentation adds to the clinical information regarding APDS, demonstrates the utility of genetic testing and illustrates the importance of a multidisciplinary collaborative approach in managing this complex syndrome.

**Supplementary Information:**

The online version contains supplementary material available at 10.1186/s13223-022-00655-5.

## Background

Activated phosphoinositide 3‐kinase (PI3K) δ syndrome (APDS) is caused by an autosomal dominant gain-of-function mutation in *phosphatidylinositol-4,5-bisphosphate 3-kinase catalytic subunit δ (PIK3CD*), which encodes PI3Kδ and is responsible for downstream intracellular signaling in leukocytes [1]. In APDS, there is increased signaling, resulting in impaired differentiation of memory B and T cells [2]. The condition is rare, with 243 known cases reported in the literature [3]. The clinical presentation includes recurrent respiratory infections, bronchiectasis, splenomegaly, lymphadenopathy, immune cytopenias, and neurodevelopmental delay, among other findings [4]. We report a child with a heterozygous de novo variant in *PIK3CD* (NM_005026.3, c.3061 G > A, p.E1021K) who, in addition to typical clinical features, was also found to have growth hormone (GH) deficiency in the setting of pituitary abnormality, nephromegaly, microscopic hematuria with proteinuria, and oligoarticular juvenile idiopathic arthritis. His clinical findings add to the available information regarding this condition and illustrate the utility of genetic testing in establishing a timely diagnosis.

## Case presentation

**Fig. 1:**
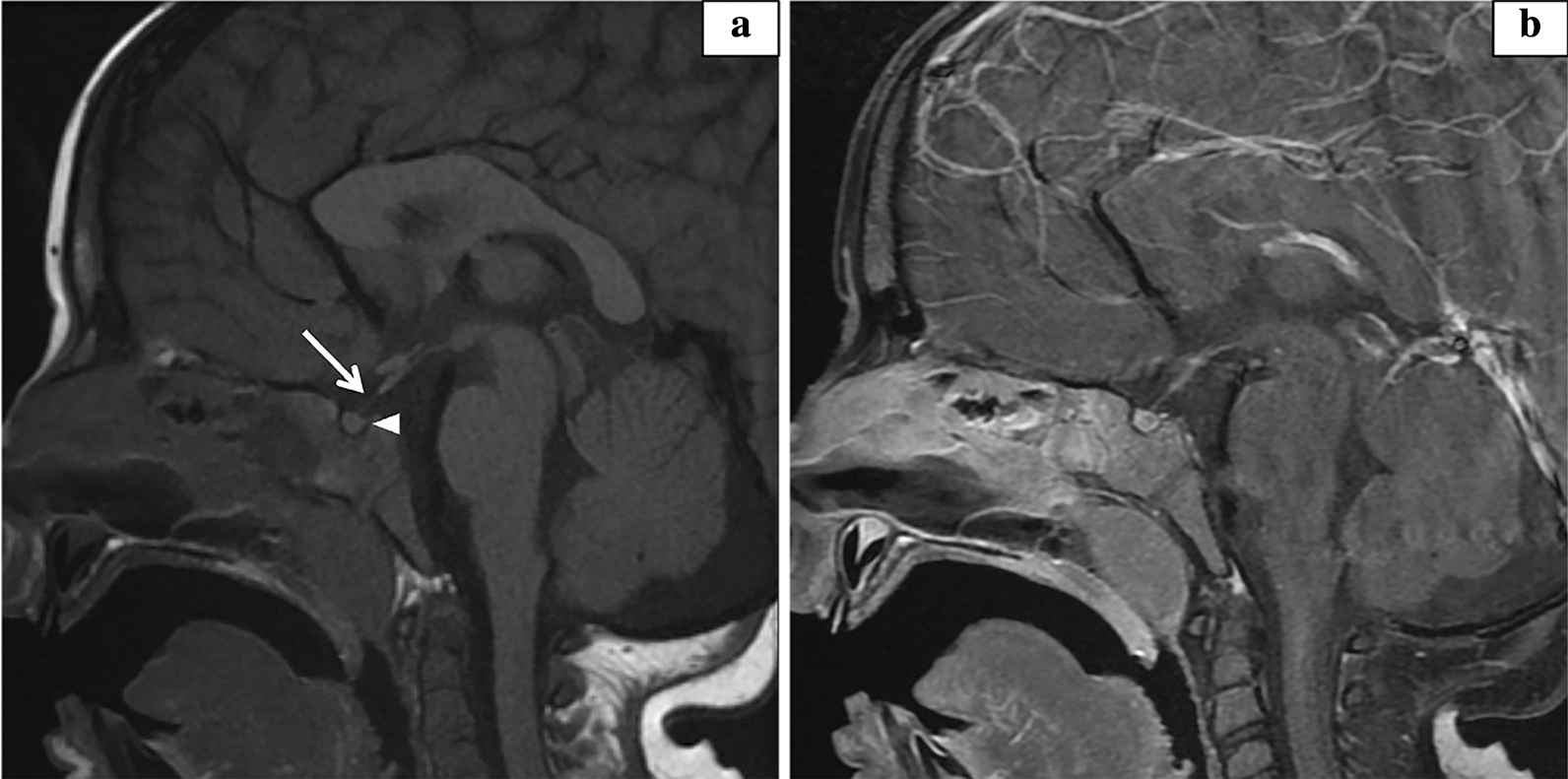
Pituitary magnetic resonance imaging. Sagittal T1 weighted images performed on a 1.5T magnet (GE Healthcare, Chicago, USA). **a** Pre-contrast T1 weighted images demonstrate nonvisualized/absent neurohypophysis (arrowhead) and abnormal thinning and hypoplasia of the mid to distal infundibular stalk (arrow). **b** Post-contrast T1 weighted images with fat suppression reveal no pituitary mass

**Fig. 2:**
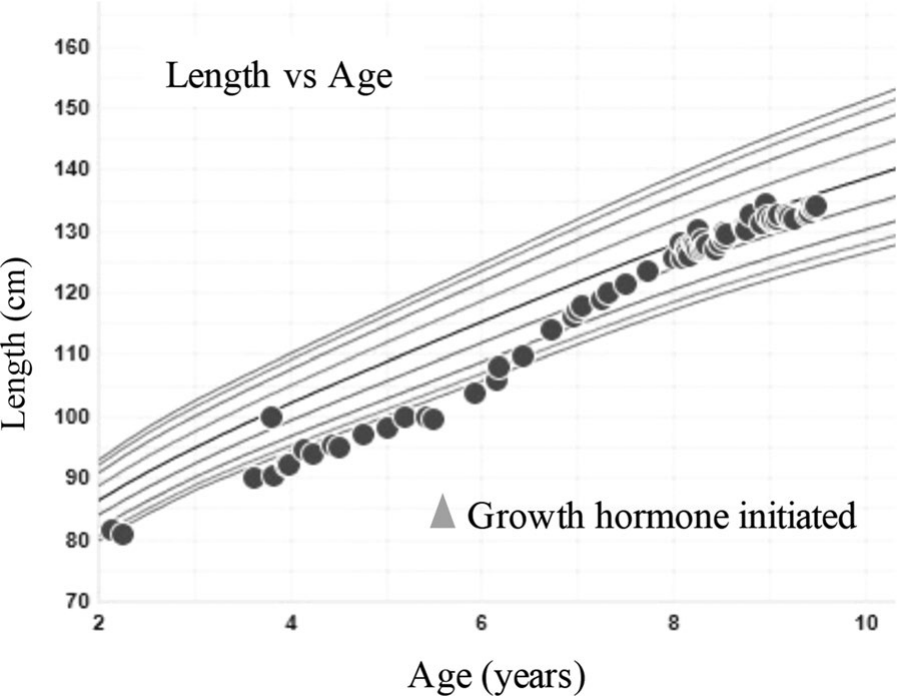
Length-for-age growth chart. Growth hormone therapy was initiated at 5 years 8 months of age (arrowhead)

A nine-year-old male presented at age five for marked growth deceleration. GH provocative testing with arginine-clonidine revealed low peak GH of 2.3 ng/mL consistent with deficiency. Subsequent pituitary magnetic resonance imaging demonstrated mildly hypoplastic adenohypophysis with thinning and hypoplasia of the infundibular stalk (Fig. 1). Laboratory evaluation for other pituitary deficiencies was negative. He started GH therapy at a dose of 0.3 mg/kg weekly with an excellent growth response (Fig. 2).

**Fig. 3:**
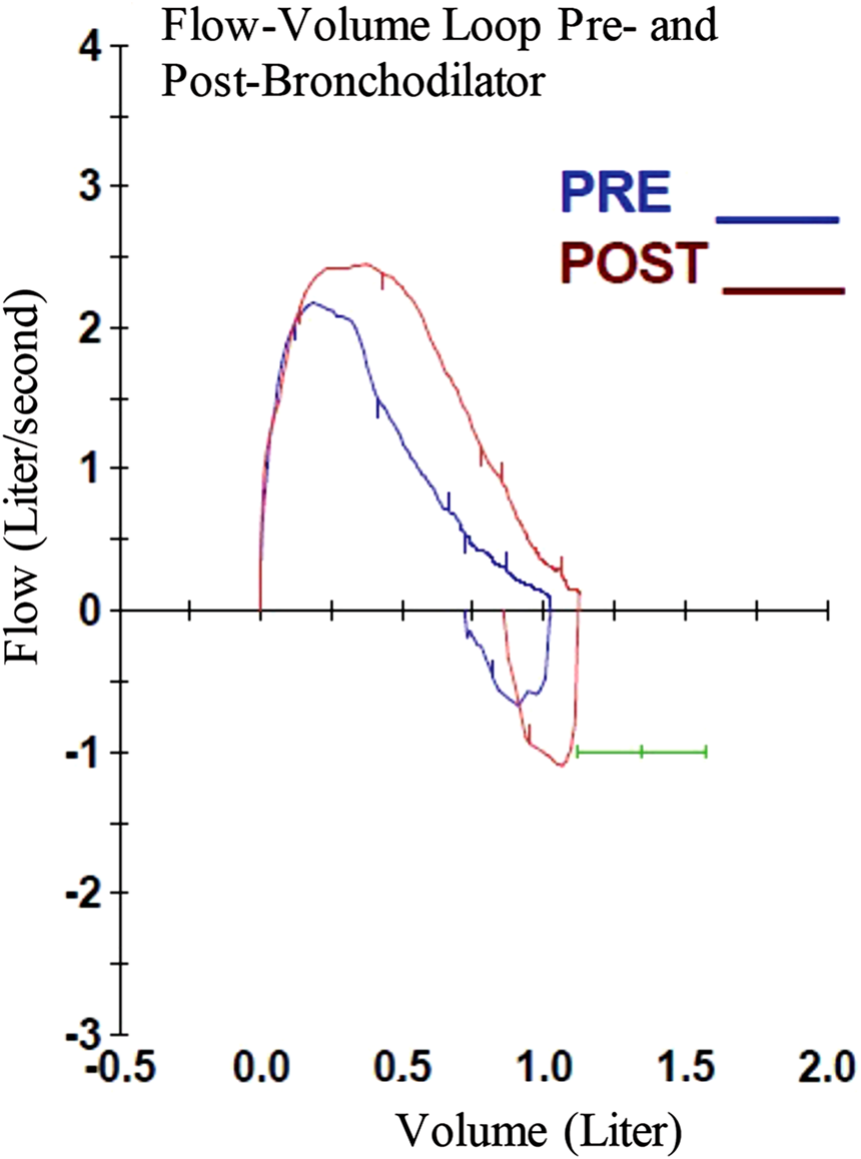
Pre- and post-bronchodilator flow-volume loop

In the next two years, he developed frequent ear and sinus infections, along with recurrent cough and wheezing triggered by viral upper respiratory infections. Wheezing episodes improved with anti-asthma medication, but the cough did not. He underwent evaluation by a pulmonologist. On examination, he was short, well-nourished and afebrile. He was macrocephalic with a normal pulmonary and cardiac examination. Initial pulmonary function tests (PFTs) were notable for a reduced and scooped expiratory loop with improvement post-bronchodilator therapy (Table 1, Fig. 3). Step-up controller therapy resulted in normalization of PFTs within 6 weeks. Nevertheless, his wet cough persisted, and the patient was later diagnosed with pneumonia. Chest radiographs after an antibiotic course showed prominent peribronchial thickening and patchy opacities in the right middle lobe. Computed tomography scan demonstrated mild bronchiectasis in the right middle lobe and reticular opacities in the right middle and upper lobes reflecting chronic changes, likely sequelae of prior infections (Additional file 1: Fig. S1). Further work up included a normal sweat test, normal airway anatomy on bronchoscopy and normal ciliary biopsy. Bronchoalveolar lavage culture grew Hemophilus influenzae. The patient was referred to immunology and genetics.

**Table 1:** Pulmonary function tests

	Pre-bronchodilator	Post-bronchodilator
FVC	1.03 L 76%	1.13 L 84%
FEV1	0.89 L 74%	1.08 L 90% post FEV1 change 22%
FEV1/FVC	86%	95%
FEF25–75	1.05 L/s 66%	1.82 L/s 74%

Pulmonary function tests with pre- and post-bronchodilator values. *FEF25–75* forced expiratory flow at 25–75% of the pulmonary volume

*FEV1* forced expiratory volume in 1 s, *FVC* forced vital capacity

His pediatrician then discovered microscopic hematuria and proteinuria on evaluation for primary nocturnal enuresis. He was referred to nephrology. Urine microscopy revealed 10 red blood cells per high power field with some being dysmorphic but no RBC casts. On initial evaluation, urine protein to creatinine ratio was mildly elevated at 0.25 mg/mg and urine albumin to creatinine ratio was also mildly elevated at 166 mg/g. Urine beta-2 microglobulin was normal. Urine cytology was benign. Electrolytes and estimated glomerular filtration rate were normal. Glomerulonephritis evaluation included: negative ANA and ANCA, normal C3, and mildly low C4 of 14. His IgA level was elevated at 464. Renal ultrasound with doppler revealed bilateral nephromegaly, with his left kidney measuring 10.6 cm (99th percentile) and his right kidney measuring 11.1 cm (100th percentile). His proteinuria appeared to worsen, particularly with exacerbations of respiratory symptoms. Proteinuria as measured on 24 h urine collection was abnormal, but not nephrotic range, at 15.5 mg/m^2^/h. A renal biopsy is planned.

At the time of his genetic evaluation at age nine, physical exam was notable for macrocephaly (Z = + 3.67) with a wide forehead and pointed chin, synophrys, protruding ears, mild gingival hypertrophy and a fused but narrow palatal vault, lymphadenopathy, and splenomegaly. There was no family history of immunodeficiency and no clear pattern of malformation on exam to account for his clinical features. In addition to exam findings and history detailed above, he had mild expressive speech delay and learning disability. There was concern for immune deficiency or primary ciliary dyskinesia given ear, sinus, and pulmonary infections requiring nine courses of antibiotics over the course of 2 years and sinus surgery with myringotomy. While some of his features including short stature, splenomegaly, facial features, and mild developmental delay, were suggestive of Noonan syndrome, the bilateral nephromegaly was considered an atypical finding. This prompted a broader evaluation for other diagnoses, including lysosomal storage disorders, common variable immune deficiency, and human immunodeficiency virus, which was negative.

Trio whole exome sequencing (WES) was pursued and revealed a heterozygous de novo missense variant in *phosphatidylinositol-4,5-bisphosphate 3-kinase catalytic subunit δ* (*PIK3CD)* (NM_005026.3, c.3061G > A, p.E1021K) the most common mutation previously reported in association with APDS [5]. Functional studies have characterized the p. E1021K variant as a gain of function mutation, resulting in increased activity of p110δ and abnormal B cell development and differentiation [6, 7]. In addition, a heterozygous paternally inherited previously reported pathogenic variant in *cystic fibrosis transmembrane conductance regulator* (CFTR) was also identified (NM_000492.4, c.3454 G > C, p. D1152H), with no second variant reported. As previous sweat chloride testing was normal, this variant was interpreted to be consistent with carrier status for cystic fibrosis.

An extensive immune evaluation was completed (Table 2). Specific antibody response testing revealed inadequate titers to 13-valent and 23-valent pneumococcal vaccine. Lymphocyte proliferation to antigens by flow cytometry demonstrated diminished proliferation to candida and tetanus toxoid in CD45 and CD3 cells. Tetanus booster did not improve his response to tetanus toxoid, which continued to show decreased T-lymphocyte proliferation on repeat testing, suggestive of impaired cellular immune memory. Flow cytometry, which can differentiate viable, apoptotic, and dead cells in a sample, revealed lower than expected viable lymphocytes. This may be due to sample collection or transport conditions; however, lymphocytes collected from patients with APDS have been shown to be more prone to cell death than lymphocytes from control groups [6]. Due to these results and the patient’s history of multiple infections requiring antibiotic treatment as described above, he began subcutaneous immunoglobulin replacement therapy and has since required only two antibiotic courses for infection in the past 13 months compared to nine courses in two years prior to initiation.

**Table 2:** Immunologic studies

Age	Study	Result	Notes
3 years 9 months	Complete blood count and differential	WBC— 19.2 TH/μL (high) Hgb— 11.0 g/dL (low) Platelets— 8 TH/μL (low) Neutrophils— 48% Lymphocytes— 35% Bands— 1% Monocytes— 1% Eosinophils— 2% Absolute neutrophil count— 9408 μL (high)	Laboratory evaluation for immune thrombocytopenic purpura
	Immunoglobulins	IgG— 1293 mg/dL (high) IgM— 206 mg/dL (high) IgA— 130 mg/dL	
	Peripheral blood smear	Severe thrombocytopenia Reactive leukocytosis with neutrophilia Slight anemia No circulating blasts	
4 years 0 months	Chronic urticaria panel	Thyroid peroxidase antibody— 3 IU/mL Thyroglobulin antibody— 75 IU/mL (high) TSH— 4.95 mIU/L (high) Histamine release- > 100% (high)	
	Complements	C3— 80 mg/dL C4— 14 mg/dL (low) C2— 1.8 mg/dL	
	Tryptase	8 ng/mL	
4 years 5 months	Chronic urticaria panel	Thyroid peroxidase antibody— 7 IU/mL Thyroglobulin antibody— 89 IU/mL (high) TSH— 2.30 mIU/L (high) Histamine release— 35% (high)	
6 years 8 months	Tryptase	9 ng/mL	
8 years 4 months	Allergen Panel	Negative for all tested allergens Total IgE— 122 kU/L (high)	
8 years 6 months	Human immunodeficiency virus (HIV)	HIV-1 antigen— not detected HIV-1 and HIV-2 antibody— not detected	
	Immunoglobulins	IgG— 1456 mg/dL (high) IgM— 282 mg/dL (high) IgA— 464 mg/dL (high)	
8 years 9 months	Trio whole exome sequencing	PIK3CD autosomal dominant c.3061 G > A p.E1021K de novo heterozygous pathogenic variant CFTR autosomal recessive c.3454 G > C p.D1152H paternally inherited heterozygous pathogenic variant	
8 years 11 months	Immunoglobulins	IgG— 1531 mg/dL (high) IgM— 301 mg/dL (high) IgA— 368 mg/dL (high) IgE— 97 mg/dL	
	Neutrophil oxidative burst	100%	
	Streptococcus pneumoniae IgG	≥ 0.30 μg/mL for 4 of 23 serotypes	Patient completed 4-dose pneumococcal conjugate vaccine series at 15 months of age
	Tetanus anti-toxoid antibody	0.52 IU/mL	The patient completed the diphtheria, tetanus, and acellular pertussis vaccine series at 4 years of age
9 years 0 months	Lymphocyte proliferation to antigens panel	Viable lymphocytes at day 0— 56.4% (low) Maximum proliferation of candida as %CD45— 1.1% (low) Maximum proliferation of candida as %CD3— 1.2% (low) Maximum proliferation of tetanus toxoid as %CD45— 0.0% (low) Maximum proliferation of tetanus toxoid as %CD3— 0.0% (low)	The patient completed the diphtheria, tetanus, and acellular pertussis vaccine series at 4 years of age These tests were completed by flow cytometry
	Lymphocyte proliferation to mitogens panel	Viable lymphocytes at day 0— 56.2% (low) Maximum proliferation of pokeweed mitogen (PWM) as %CD45— 7.0% Maximum proliferation of PWM as %CD3- 8.5% Maximum proliferation of PWM as %CD19— 3.5% (low) Maximum proliferation of phytohemagglutinin (PHA) as %CD45— 66.7% Maximum proliferation of PHA as %CD3— 75.4%	
	T- and B-cell quantitation	CD45 total lymphocyte count— 2.29 TH/μL CD3 T cells— 70%, 1593 cells/μL CD4 T cells— 28% (low), 648 cells/μL CD8 T cells— 40% (high), 922 cells/μL CD19 B cells— 21%, 485 cells/μL NK cells— 8%, 190 cells/μL 4.8 ratio— 0.7 (low)	
	Peripheral blood smear	Mild leukocytosis with mild relative and absolute neutrophilia Relative lymphopenia Slight anemia	
9 years 2 months	Complete blood count and differential	WBC— 14.7 TH/μL (high) Hgb— 11.2 g/dL (low) Platelets— 230 TH/μL Neutrophils— 74% (high) Lymphocytes— 16% (low) Monocytes— 8.6% (high) Eosinophils— 0.8% Absolute neutrophil count— 10.91 TH/μL (high)	
	Lymphocyte proliferation to antigens panel	Viable lymphocytes at day 0— 50.9% (low) Maximum proliferation of candida as %CD45— 2.8% (low) Maximum proliferation of candida as %CD3— 4.2% Maximum proliferation of tetanus toxoid as %CD45— 0.1% (low) Maximum proliferation of tetanus toxoid as %CD3— 0.1% (low)	The patient received the tetanus, diphtheria, and acellular pertussis vaccine 5 weeks 6 days prior These tests were completed by flow cytometry
	Lymphocyte proliferation to mitogens panel	Viable lymphocytes at day 0— 50.9% (low) Maximum proliferation of PWM as %CD45— 5.7% Maximum proliferation of PWM as %CD3— 7.6% Maximum proliferation of PWM as %CD19— 3.0% (low) Maximum proliferation of PHA as %CD45— 54.2% Maximum proliferation of PHA as %CD3— 67.6%	
	T- and B-cell quantitation	CD45 total lymphocyte count— 2.11 TH/μL CD3 T cells— 68%, 1431 cells/μL CD4 T cells— 30%, 624 cells/μL CD8 T cells— 37% (high), 781 cells/μL CD19 B cells— 24%, 499 cells/μL NK cells— 8%, 162 cells/μL 4.8 ratio- 0.8 (low)	
	Streptococcus pneumoniae IgG	≥ 0.30 μg/mL for 12 of 23 serotypes	Patient received pneumococcal polysaccharide vaccine nine weeks prior

Chronologic list of immunologic studies

*HIV* human immunodeficiency virus,* PHA* phytohemagglutinin, *PWM* pokeweed mitogen

Shortly after his molecular diagnosis at age nine, the patient was admitted for worsening acute on chronic left hip pain. On exam, skin was intact, without associated redness, swelling or tenderness of the left leg. Hip flexion and external rotation were normal but groin pain limited range of motion with internal rotation (15 deg), which manifested as an antalgic gait. Laboratory studies revealed mild leukocytosis with normal inflammatory markers. MRI was notable for moderate left hip effusion with synovial enhancement, moderately prominent inguinal and iliac lymph nodes, and no avascular necrosis. He underwent arthrocentesis and diagnoses of aseptic versus inflammatory arthritis was considered. Symptoms resolved spontaneously soon after the procedure. Rheumatology was consulted and diagnosed oligoarticular juvenile idiopathic arthritis based on history and imaging findings; no abnormalities in the musculoskeletal exam were noted at the time, and he was started on naproxen for symptomatic management.

## Discussion and conclusions

Our patient has an autosomal dominant gain-of-function amino acid substitution of glutamic acid for lysine on PI3Kδ’s catalytic subunit, P110δ [6]. This change induces phosphorylation of downstream messenger phosphatidylinositol 3,4,5-trisphosphate at up to six times the wildtype rate [6]. Clinical findings associated with this specific mutation include recurrent respiratory and ear infections, large or small airway disease, splenomegaly, abscess formation, cellulitis, susceptibility to herpes group viruses infections, and marginal zone lymphoma [6]. Immunologic features include low anti-pneumococcal antibodies [6], as seen in our patient, among other findings. This patient exhibits a unique profile of clinical features, including aseptic arthritis, growth hormone deficiency, asthma, bronchiectasis, specific antibody deficiency, nephromegaly and microscopic hematuria and proteinuria that expand the phenotypic presentation of this mutation.

Asthma, as seen in our patient, has been previously reported in association with this condition [3]. PIK3δ regulates cellular response to vascular endothelial growth factor, an important mediator of vascular leakage and thus inflammation [8]. It is known that PI3Kδ expression is elevated in bronchial biopsies of patients with asthma compared with healthy controls, and PI3Kδ inhibitors have been shown to suppress cytokine release from these cells [9]. An oral inhibitor of PI3Kδ has even been suggested for therapeutic management of patients with asthma [9, 10]. Based on these reports, it is reasonable to conclude that overactive PIK3δ may increase bronchial inflammation, leading to our patient’s clinical findings.

Nephromegaly has not been noted previously in association with APDS. However, glomerulonephritis and lupus-like nephritis have previously been described [3]. There was one case of a child with PIK3δ who also met criteria for systemic lupus erythematosus based on persistent proteinuria and hematuria, among other findings [11]. In a murine model, PI3Kδ inhibition reduced macrophage infiltration of the kidney by diminishing the macrophage’s ability to cross the basement membrane [12]; if PI3Kδ were overactive, macrophages may infiltrate renal tissue more readily, leading to nephromegaly. In our patient, testing for storage disorders, common variable immune deficiency, human immunodeficiency virus, and Noonan syndrome was negative. He has not yet undergone kidney biopsy so the mechanism of nephromegaly in his case remains uncertain. However, worsening of his proteinuria with respiratory infections could indicate IgA nephropathy or other immune complex glomerulonephritis.

Rheumatologic disease, including juvenile idiopathic arthritis, is associated with APDS. PI3Kδ is expressed at higher levels in the synovium and synoviocytes of patients with rheumatoid arthritis than in patients with osteoarthritis and has been shown to regulate migration and invasion of fibroblast-like synoviocytes [13, 14]. Increased migration and invasion of these cells may be the etiology of increased arthritic disease in APDS patients. In general, APDS is associated with immune dysregulation, which may play a role in this process.

Some of the clinical features found in our patient have been reported in patients with the related disease, APDS2, which is caused by a gain-of-function mutation in the gene PI3K regulatory subunit one that encodes a regulatory subunit [15]. Children with APDS2 have also been reported to have short stature and poor growth, sometimes even receiving GH replacement [15]. However, GH has been reported to exacerbate lymphoproliferation as evidenced by increased lymphadenopathy [16], likely due to the role of GH in signaling via the PI3K pathway [17]. Our patient’s diagnosis prompted concern that GH therapy could induce further lymphoproliferation and thereby increase risk of malignancy, which is seen in about 12% of patients with this condition [3]. However, he was allowed to continue as the pathway was considered constitutively active at baseline and he had an excellent growth response to GH over three years prior to his genetic diagnosis. Pituitary abnormalities as seen in our patient have not been reported and may represent a novel finding related to APDS.

This patient’s unique findings add to the clinical information available for this rare form of primary immunodeficiency and illustrate the importance of a multidisciplinary collaborative approach in managing this complex syndrome. WES was instrumental in obtaining this patient’s timely diagnosis once his array of clinical findings raised concern for an underlying genetic disorder.

## Supplementary Information

**Additional file 1: Figure S1. **Computed tomography of the chest without contrast reveals minimal bronchiectasis within the medial segment of the right middle lobe. Performed on a Dual Source SOMATOM Force CT Scanner (Siemens Healthineers AG, Melvern, PA). **a** Coronal view with bronchiectasis (arrow). **b** Axial view with bronchiectasis (arrow).

## Data Availability

Not applicable.

## Abbreviations

APDS: Activated phosphatidylinositol-4,5-bisphosphate 3-kinase δ syndrome
CFTR: *Cystic fibrosis transmembrane conductance regulator*
FEF25-75: Forced expiratory flow at 25–75% of the pulmonary volume
FEV1: Forced expiratory volume in 1 s
FVC: Forced vital capacity
GH: Growth hormone
HIV: Human immunodeficiency virus
PFT: Pulmonary function test
PHA: Phytohemagglutinin
PI3K: Phosphoinositide 3-kinase
*PIK3CD*: *Phosphatidylinositol-4,5-bisphosphate 3-kinase catalytic subunit δ*
PWM: Pokeweed mitogen
TH: Thousand
WES: Whole exome sequencing
