# Supplementary figures and images for: Activated phosphoinositide 3-kinase δ syndrome associated with nephromegaly, growth hormone deficiency, bronchiectasis: a case report
Source: Allergy Asthma Clin Immunol. 2022 Feb 21;18:15. doi: 10.1186/s13223-022-00655-5 (PMC8862239; doi:10.1186/s13223-022-00655-5)

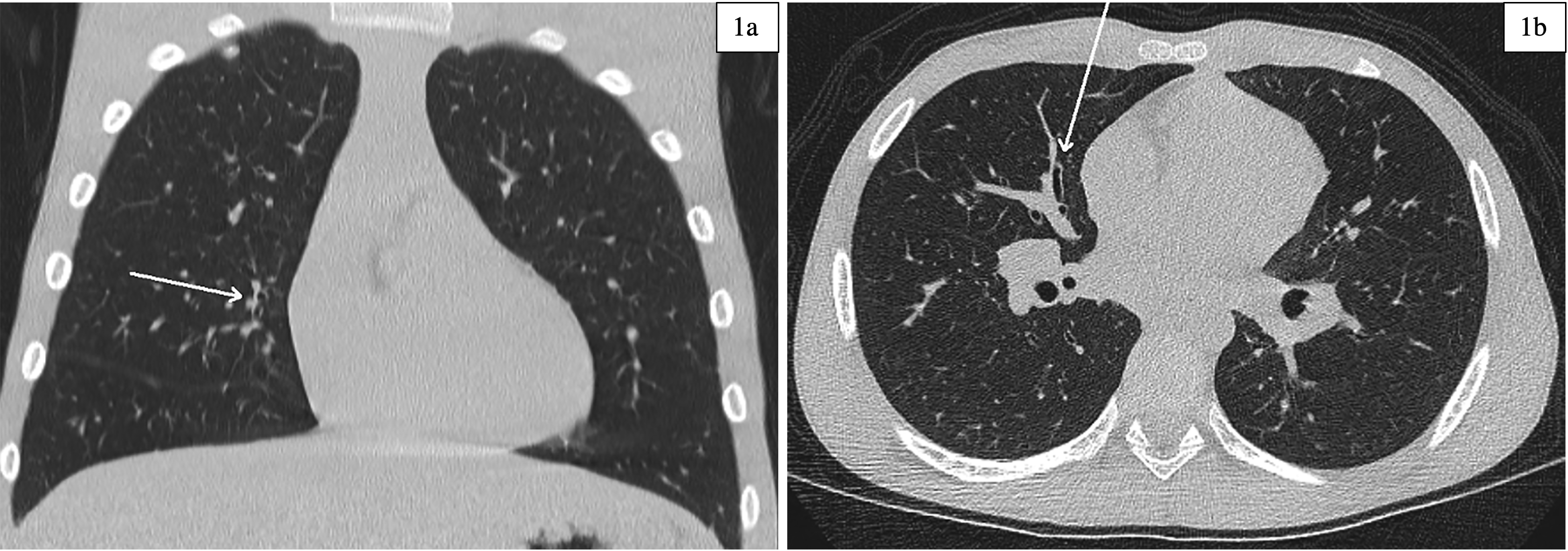

Supplement: Supplementary file 1 — Additional file 1: Figure S1. Computed tomography of the chest without contrast reveals minimal bronchiectasis within the medial segment of the right middle lobe. Performed on a Dual Source SOMATOM Force CT Scanner (Siemens Healthineers AG, Melvern, PA). a Coronal view with bronchiectasis (arrow). b Axial view with bronchiectasis (arrow). [file 13223_2022_655_MOESM1_ESM.tif]
